# Selective Modifications of Sheep Wool Usable in Non-Textile Applications

**DOI:** 10.3390/polym16101380

**Published:** 2024-05-12

**Authors:** Jana Braniša, Karin Koóšová, Mária Porubská

**Affiliations:** Faculty of Natural Sciences and Informatics, Constantine the Philosopher University in Nitra, Tr. A. Hlinku 1, 949 01 Nitra, Slovakia; jbranisa@ukf.sk (J.B.); karin.koosova@ukf.sk (K.K.)

**Keywords:** sheep wool, modification, scouring, dyeing, antifelting

## Abstract

The traditional textile use of wool as a valuable renewable material needs alternative applications in order to, besides sheep milk and meat, valorize currently unnecessary wool. Each type of product containing sheep wool requires wool with customized properties. Finding suitable physical and chemical modifications needed to develop new products while minimizing harmful side effects is a challenge for scientists. The presented review provides a brief overview of works published over the last decade associated with innovative wool scouring, dyeing, antifelting, and modification of its structure without the ambition to present complete, detailed data.

## 1. Introduction

Many biomaterials offer amazing properties that have been underappreciated or used one-sidedly for years. Among them is also sheep wool, which was mainly used for textile production in ancient times. As research has gradually shown, the chemical and physical structure of wool is a perfect work of nature. Relevant detailed data on the structure with illustrations can be found in the review in ref. [1].

**Figure 1:**
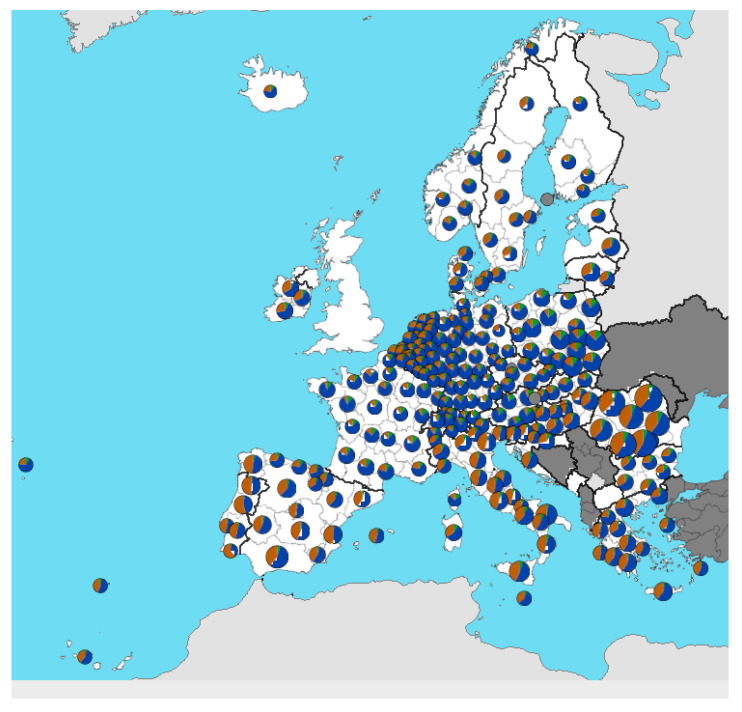
Areas of the EU where sheep are bred [2]. The different coloring of the points shows the percentage age structure of farm managers (<35 years—green, 35–64 years—blue, ≥65 years—brown).

Traditional sheep breeding is an important component of agricultural production in every country where the natural conditions are suitable. Sheep are bred in every European country. Eurostat (excluding England) states [2] that, in 2022, pastures in Europe occupied more than 58% of locally utilized agricultural area in countries including Ireland, Iceland, Spain, France, Switzerland, Austria, Montenegro, North Macedonia, Greece, and Turkey. The countries Portugal, Belgium, Netherlands, Luxembourg, Germany (Saarland), Slovenia, Romania, Bulgaria, and Italy (Sicily) used 42–58% of the agricultural area for sheep breeding. Less than 12% of the area was occupied by pastures in Hungary, Serbia, Sweden, Denmark, and Finland. The localization of breeders can be seen in Figure 1.

Sheep meat and milk are used in the food industry, while wool is a raw material mainly used in textile products. The development of synthetic fibers greatly suppressed the demand for wool, as a result of which, alongside the profitable milk and meat, wool became a loss-making item. In addition to the costs of necessary sheep shearing, the more difficult marketable wool must be stored and disposed of as hazardous animal waste. Following the European Commission regulations [3] on animal by-product control, unserviceable raw wool is classified as a category 3 special waste material. The collection, storage, transport, treatment, use, and disposal of such unserviceable raw wool are subject to European Union regulations because of a potential risk to human and animal health. Thus, the legal disposal of wool waste increases the costs for breeders even more. The EU’s stricter environmental protection rules have caused the partial liquidation of wool washing plants that proved unprofitable; alternatively, they moved to countries with more benevolent legislation. In addition, globalization has led to the atrophy of the textile industry in several European countries. However, improving the profitability of sheep farming is desirable from a food, ecological, and social point of view.

These facts logically stimulated the search for new ways of using wool, especially coarse wool and lower-quality wool. The ingenious structure of wool keratin has attracted the attention of scientists. Although research on the innovative use of this renewable resource compared to textile applications is only at an early stage, studies on various physical and chemical treatments of wool can already be found in information databases. Such products can be used in the consumer industry, agriculture, construction, environmental protection, cosmetics, and healthcare.

The presented study aims to briefly summarize the works published in scientific databases, mainly in the recent period, focused on the physico-chemical treatment of sheep wool associated with wool scouring, dyeing, antifelting, and modification of its structure. This selection does not cover all areas of wool use, as there are few, if any, areas of human activity where wool and related products are not found.

## 2. Keratin Structure in Brief

**Figure 2:**
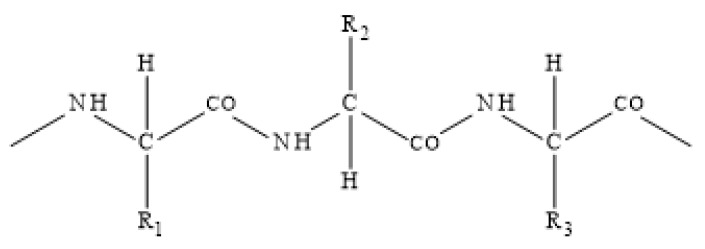
Simplified scheme of the keratin protein chain.

The basis of sheep wool is the biopolymer keratin. The primary chemical structure of the fiber is determined by a protein basis with a certain sequence and type of amino acids, which are connected to each other by peptide bonds. Keratin is an ampholyte, i.e., a substance that behaves depending on the pH of the environment as both an acid and a base, which describes the short notation of keratin molecule as NH_2_-wool-COOH. Cross-linking of peptide chains by R-S-S-R disulfide bridges ensures the insolubility of wool in water. A simplified formula of keratin macromolecule with side groups R_1,2,3_ is shown in Figure 2.

Keratin chains can be grouped into a three-dimensional right-handed α-helical conformation or a planar β-sheet conformation, always with some proportion of an amorphous portion. During felting, a more contracted meander-like arrangement of γ-keratin is also formed. The surface of the fiber is covered with wax, and lanolin, which is intimately connected to the fiber and gives it a hydrophobic character. The outer layer of the fiber consists of scales, and cuticle, being oriented in one direction and partially overlapping each other.

The quality of the fiber is influenced by the breed of sheep, nutrition, and climatic conditions [4,5]. In general, it can be said that females and lambs have better quality, finer wool. According to the biophysical conditions, the thickness of the fiber changes. Geographical location and season play a role, too.

Sheep wool contains four (O, C, H, N) of the five primary biogenic elements. The building keratin units are made up of 20 amino acids. However, complete acid hydrolysis of wool yields a mixture containing only 18 amino acids, because during acid hydrolysis asparagine and glutamine are converted to the corresponding acids [6,7].

## 3. Scouring Wool

Any purpose of using wool, except for its targeted destruction, e.g., for compost, requires scoured wool. The task of the scouring is to remove not only dirt but also the hydrophobic layer of surface lipids, which inhibit the wetting of wool necessary for further technological steps. This initial part of the processing procedure is often the most demanding procedure. Mechanical stirring of soiled wool in a hot water neutral or slightly alkaline bath containing a non-ionic detergent always leads to felting of the wool. Mechanical stirring of dirty wool in a hot neutral or slightly alkaline bath containing a non-ionic detergent always leads to felting of the wool. Therefore, it is not surprising that in addition to traditional scouring technologies producing a lot of polluted wastewater, innovative efforts are also appearing here. A promising method appears to be the use of ultrasound (US) [8,9], which could reduce costs, environmental burden, and felting of scoured wool.

The principle of the US cleaning effect is cavitation, i.e., the formation of a huge number of microbubbles—hydrodynamic cavities in water caused by high-frequency oscillation of a resonator and accompanied by a precipitous creation of a transient vacuum. The subsequent implosion of the microbubbles causes a shock wave capable of tearing off microparticles of dirt from the fiber. Several researchers focused on the use of this principle and investigated the possibilities of scouring wool with the support of US methods [8,9,10,11]. The experiments were carried out on a laboratory scale (Table 1), but no information is known about the transfer of knowledge to industrial practice in the sense of building a functional ultrasonic scouring mill.

**Figure 3:**
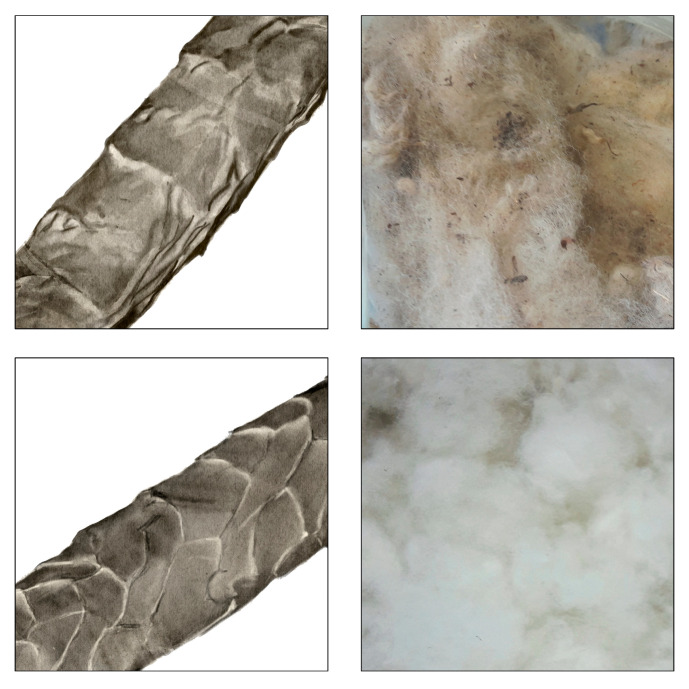
The surface appearance of sheep wool before (**up**) and after (**down**) scouring in an ultrasonic bath (Adapted by Z. Braniša from [9]).

The industrial application of US methods must be preceded by a thorough analysis of all related aspects, such as e.g., suitable design of the device, the effect of ultrasound frequency, bath temperature, time of US action on the structure and properties of the wool fiber itself, the efficiency of dirt removal (such as manure, plant residues, and grease), etc. The appearance of a fiber scoured using US methods is displayed in Figure 3.

Since most authors focus on the following wool dyeing process after washing, they evaluate the effect of US washing according to the quality of dyeing of the wool cleaned in the US bath. Li et al. [8] investigated the effect of US frequency and power on process performance with varying liquor ratios. It was found that the purity and yellowness of the wool was not significantly affected by US energy. On the other hand, an effect of frequency was observed; when applying 28 kHz and 80 kHz, more grease and dirt were removed than at 45 kHz. The effect of US methods was manifested by cracking to peeling of the scales, which was more ascertained when scouring at low US frequencies. Disruption of the fiber cuticle facilitated dye uptake. Similar conclusions were reached in the study of Pan et al. [9], when during acid and reactive dyeing at 40 kHz and 80 kHz, color strength (i.e., ability to impart color to other materials) was significantly better compared to conventionally dyed wool.

The findings of Nazmul Islam et al. [10] about the impact of US methods complement the conclusions of the above-mentioned authors [8,9]. Samples of woolen knitted fabric were dyed with acid dye without and using ultrasound with (30 kHz) at 60, 70, and 80 °C. Applying the US frequency of 30 kHz, no damage to the fiber surface was observed; however, the US method increased the rate of dye uptake. The authors explain this fact by a reduction in the viscous boundary layer of liquor around the fiber caused by US methods, which facilitate dye migration into the fiber even at lower temperatures compared to conventional dyeing at 98 °C without US action [10].

Braniša et al. [11] compared wool scouring using three parallel procedures consisting of (i) a US (38 kHz) tempered bath 5 L in volume with 40 °C tap water, during 2 × 10 min with replacement of the used water, (ii) the same as (i) but with commercial detergent intended for wool fabric, and (iii) 4 h dichloromethane extraction with 14 drain-off cycles. The scouring efficiency criteria used were weight loss and adsorptive removal of Cu, Zn, and Pb cations from model solutions. Statistic testing of the obtained data showed consistency for all weight losses, but metal adsorption varied slightly, as expected, depending on the chemical properties of the tested cations. Regardless, it was concluded that US wool scouring in only warm water without any chemicals is satisfactory (Figure 3) and favored because it did not use either carcinogenic organic solvent or detergents, and thus it is more environmentally friendly. In addition, the used water is not wasted, but can serve as a complementary fertilizer of the soil. Czaplicki et al. [12] used a two-step scouring procedure. In the first phase, feces were removed from the wool in a water bath. In the second scouring phase, a special apparatus with a US 40 kHz generator was used. The US bath contained detergent, alkali soap, and sodium carbonate. The optimal scouring time, detergent concentration, and bath liquor ratio to wool mass were tested in order to achieve the best dirt removal results.

Valuable information on the influence of US bath temperature on the wool dyeing process was provided by the study of Actis Grande et al. [13]. It used both theoretical and experimental approaches and their comparison applied a simplified mathematical model of the implosion of a simple bubble. It was shown that the “maximum implosion pressure” obtained by calculation from the Rayleigh–Plesset equation can be correlated with the cavitation intensity experimentally measured with an ultrasonic energy meter. The correlation of the theoretical data calculated from the implosion pressure with the experimental data was satisfactory. The results indicated that in the temperature range of (50–60) °C, the intensity of cavitation is almost constant and still sufficient. This validated the results of US dyeing previously obtained at low temperatures of (40–70) °C. The comparison of coloring with and without the US exposure was verified by standard coloristic tests ΔE (color variation), Re, % (reflectance percentage), K/S (color strength), and color fastness. The results were compared with conventional washing at 98 °C. It was found that for US-assisted dyeing, it is appropriate to choose a temperature of around 60 °C, which is a compromise between the intensity of cavitation and the kinetics of dye diffusion into the fibers. Cavitation effects decrease rapidly by increasing the temperature and the cavitation intensity at lower temperatures was quite high.

Pan et al. [14] investigated the effect of US on wool fiber morphology, physico-mechanical characteristics of the scoured fiber, and its dyeability. The experimental scouring was performed in a 28 kHz US bath of 10 L in volume, at 45/80 kHz frequency, a maximum power of 300 W adjustable from 40% to 100% power, and an adjustable bath temperature between 20 and 80 °C. Five scouring cycles lasting 3 min each using a bath liquor ratio of 1:100 were used. Scanning Electron Microscopy (SEM) images of the fiber surface scoured at a low frequency of 45 kHz showed severe cuticle damage. Despite this, the effect on mechanical properties was negligible, especially on bending abrasion resistance. The effect on the dyeability of ultrasonically scoured fibers was tested using both acid and reactive dyes. As observed during the early stage of dyeing, ultrasonically scoured wool has a quicker dye uptake at low temperatures up to (60–65) °C for acid dye, and (50–55) °C for reactive dye, than the conventionally scoured samples.

Since the traditional scouring agents are soda ash and various detergents, the final scouring operations involve multiple rinsing of the wool with water to remove alkali, which is associated with high water consumption. Taleb et al. [15] focused on researching the possibility of using immobilized thermophilic lipase to remove the lipid layer instead of traditional chemicals. The necessary thermostable lipase was prepared from a thermophilic microorganism (*Bacillus aryabhattai B8W22*), whose laboratory preparation and characterization included several laborious steps. The produced enzyme was immobilized on sericin-based discs. The prepared enzyme in free and immobilized form was each individually applied in one scouring bath, which was subsequently also a dye bath. Combining both operations into one bath saved water consumption. Both the scouring and dyeing phases were conducted under different conditions (temperature, pH, concentration, and time). The resulting properties of the washed and dyed wool samples were monitored using SEM, UV/Vis spectrometry, chemical analysis, and bundle strength. As a result, for bioscoured samples, dye exhaustion was enhanced so that dyeability was better in comparison with classic scouring. The structure of the wool surface was not disturbed by bioscouring and the immobilized lipase can be reusable for up to five bioscouring cycles at room temperature and pH 6. In order to decide on the profitability of the method used, it is necessary to calculate the costs for the laborious preparation of thermostable lipase and compare them with the savings in water consumption.

## 4. Anti-Felting and Anti-Shrinking Treatment

Wool felting is a process in which the fibers are entangled with each other by mechanical and thermal stress due to their scaly surface. The fiber scales are oriented in one direction, similar to the scales of a spruce cone. When fibers are forced to move over the surface of other fibers with the opposite orientation of the scales, they become caught and entangled with each other. In many technological steps and applications of sheep wool, felting is undesirable. It is known that woolen clothes require particularly careful washing in order to avoid felting, shrinkage, and even deterioration of the garment. Standard scouring of raw wool in a wool scouring mill is always associated with a certain degree of felting, and therefore the scoured and dried wool must be combed.

Iglesias et al. [16] were the first to deal with the possibility of limiting wool felting during scouring using biosurfactant. They applied an eco-friendly anti-felting treatment of wool top based on biosurfactant *Bacillus O9* biosurfactant (surfactin), and enzymes extracellular proteolytic extracts from *Bacillus* sp. *G51* and *Bacillus patagoniensis PATO5^T^*. The effect of the proteolytic enzymes was tested individually as well as in combination with the biosurfactant. The results of the felt-ball test indicated that a significant decrease in wool felting was achieved with the usage of 50 and 150 Enzyme Units (EU)/g wool of *PATO5T* and *G51 proteases*, respectively. It is favorable that the tensile strength was not significantly reduced, when the control sample was (50.1 ± 1.7) N/kTex, while wool treated with proteases *PATO5^T^* showed (46.2 ± 2.2) N/kTex. If the fibers were first treated in a bath of biosurfactant with a concentration higher than the critical micelle concentration (20 mg/L) before the action of the enzymes, the tendency to felting was further reduced. In addition to the excellent properties of wool, the irreversible shrinkage of woolen clothes during washing is a serious drawback. It limits the washing of textiles in the washing machine. This issue is addressed in a review study by Hassan and Carr [17]. They mention oxidative chlorination of wool tops using gaseous chlorine, sodium hypochlorite (NaOCl), and dichloroisocyanuric acid (DCCA) as anti-shrinking treatment procedures. The chlorination removes the surface-bound 18-methyl eicosanoic acid from wool fibers and also etches the edge of wool fiber scales. As stated by [17], ozone (O_3_) in the acidic medium can also be used for oxidation to avoid strong damage to tensile properties. Enzymatic degradation of scales on wool fiber surfaces can also provide shrink resistance. A similar result can be achieved by radiation from various sources such as ultraviolet, gamma, X-ray, infrared, and radio waves, which are used for the chemical modification of fibers and polymers. Radiation ionizes or excites the atoms or molecules in fibers and produces hydroxyl radicals. These can cause degradation in keratin chains to make wool fiber shrink-resistant. Plasma being a mixture of partially ionized gas and generated by an electrical discharge is also applied to obtain dimensional stability of wool. A certain disadvantage is that plasma treatment is a discontinuous process. The biodegradability of biopolymer coatings must also be taken into account when developing a shrink-resistant treatment for wool [17].

Another method of improving the dimensional stability of wool fabric was investigated by Rani et al. [18]. Scoured wool fabric was immersed for 10 min in a mixed solution of biopolymers composed of gum arabic, chitosan, and wheat starch. After that, the soaked material was passed through rollers to prepare a pad of uniform thickness, which was dried in an oven at 60 °C and then cured at 145 °C for 10 min. After being rinsed with water, the sample was freely dried. Control of the biopolymer’s presence was performed using FTIR and SEM. The properties of treated samples were measured, namely moisture, tensile, frictional, and bending properties. Moisture content (≈13.5%) and moisture regain (≈15.5%) of all samples were comparable with the untreated wool. However, chitosan treatment showed increasing the tensile strength and tensile modulus (by 13% and 17%, respectively) and, the strain was the same as the reference sample (23.5%). A more significant growth was found for flexural rigidity and bending modulus (both by 44%). While no adverse impact was found on the other fabric characteristics in comparison with non-treated fabric, the area shrinkage was significantly reduced (<4%) of the modified wool fabric. In another work, Kadam et al. [19], prior to the application of the above-mentioned biopolymers on wool, pre-treated the action of the enzyme laccase in one group of samples and protease in another group. The intention was enzymatic cleavage of the cuticle to improve the coating of the fiber with biopolymers. The enzymes were then deactivated by immersing the treated samples in hot 85 °C water. The next procedure was the same as in previous experiments [17] with the difference that the curing temperature was 120 °C. While the area shrinkage for the unmodified fabric was measured as 16.4%, the combined treatment showed only 3.6%. A reduced yellowness of the modified fabric was also found, which may be an advantage compared to conventional chlorine bleaching.

The permanent interest in the enlarging of eco-friendly techniques for reducing shrinkage in the finishing of dyed wool is obvious. Li et al. [20] tried to hydrolyze wool scales by mature protease *Savine 16L*. However, the protease easily penetrated the cell membrane complex (CMC), which caused undesirable damage to the fiber including physical properties. To limit this effect, they used a combination with cyclamate, which made the protein molecule larger, limiting its mobility and making its influence on intermolecular forces more difficult. Under these conditions, the effect of the protease was redirected to the scales. By applying keratinase during the pre-treatment of the wool, the disulfide bonds in the scales were hydrolyzed, thereby releasing the integrity of the wool structure. Thus, the combination of keratinase, protease, and cyclamate made the wool more resistant to shrinkage, which reached 5.4%. The strength loss was 12.3%, which is acceptable. The color shade of the woolen fabric was almost unchanged compared to the chlorination method.

## 5. Wool Dyeing

The quality of wool dyeing is an important factor from an aesthetic point of view. In the fashion industry, it is important to design clothing according to the latest trends, usually with prints. On woolen fabric, it is difficult to achieve a printed pattern with sharp contours and vivid colors due to scales on the surface of the fibers. Experiments conducted in order to improve the printing result were carried out by An et al. [21]. They combined the effect of the protease enzyme and sodium alginate. Microscopic images revealed that the protease removed most of the scales and the alginate covered the newly formed surface with a homogeneous film with improved hydrophilicity documented by a reduction in contact angle and a reduction in wetting time. X-ray photoelectron spectroscopy (XPS) analysis showed a higher content of C, O, N, and less S in the treated samples compared to the untreated sample. The effect of fiber treatment was tested by printing using magenta, yellow, and black reactive dyes. As shown by the quantification of the color parameters (CIEL*a*b*; color strength K/S), the quality of the printed color patterns increased in the order as follows: untreated wool < treatment with protease only < treatment with alginate only < combination of protease and alginate. The synergistic effect of protease and alginate maintained satisfactory washing fastness, abrasion resistance, and strength.

An alternative method of improving the color printing of woolen fabrics with reactive wool dyes was examined by Wang et al. [22]. They used compressed air plasma to etch the surface scales of the cuticle. The enlarged surface was treated by sizing with a mixed aqueous solution of urea, sodium alginate, and ammonium sulfate. As found out by Barani and Haji [23], treatment with oxygen plasma transformed the α-helical arrangement of keratin to the β-pleated sheet configuration. Better conditions for the absorption of dye inks by fibers and the expansion of binding points after breaking C-C and C-N bonds facilitated the formation of covalent bonds between the fiber and the tested dyes R-218 and 0–13, which was confirmed by standard coloristic tests listed above [21]. In follow-up work, Hassan et al. [24] included in the single-step synthesis of dye also wool textile coloration. The dye was synthesized by the mechanism of oxidative polymerization of non-toxic monomers 7-amino-1,3-naphthalene monopotassium disulfonate, 4-amino-1-naphthalene sulfonic acid, and 5-amino-2-naphthalene sulfonic acid. The authors expanded the coloristic testing methods with gel permeation chromatography (GPC) and mass spectrometry to control the resulting polymers. The GPC results indicated that all monomers polymerized and their GPC parameters were very close. In this case, too, it was shown that variations in parameters such as monomer type, oxidant concentrations, treatment pH, time, and temperature affected the color characteristics of the dyed fabric. The position of the sulfonate groups in the amino-naphthalene compounds determined the shade and the color intensity of the color produced. Increasing the temperature and time of treatment increased the color fastness of the fabric. The optimum conditions were 50 °C, 120 min, and a monomer/oxidant ratio of 1:1. A comparison of energy consumption under these conditions with the traditional dyeing with acid dyes at the boil shows almost a 48% reduction of the energy consumption.

Improvement of the dyeing process by simple grafting of pre-synthesized chitosan-acrylamide hybrid onto wool in the presence of citric acid as a cross-linking agent was reported by Sadeghi-Kiakhani et al. [25]. The result of grafting was confirmed by FTIR, SEM, X-ray diffraction analyses, and weight growth techniques. The comparison of the dyeing of raw and grafted wool after the application of reactive dyes Reactive Red 195 and Reactive Black 5 was performed using the CIEL*a*b* coloristic method and K/S, indicating the depth of color of a dyed fabric. The results of chitosan–acrylamide-grafted wool showed better parameters compared to raw wool; the temperature of the dye bath, the required dyeing time, and the consumption of the reactive dye were lower with the modified wool and a deeper color shade could be achieved. Moreover, the modified wool showed very good radical scavenging and excellent antibacterial activity against gram-negative (*E. coli*) and gram-positive (*S. aureus*) bacteria. Therefore, the mentioned modification has favorable economic and also environmental and biological effects.

The process of dyeing wool with acid dyes usually takes place under boiling. The possibility of reducing the related energy costs was investigated by Hassan [26] using an inverse approach focused not on wool modification but on dye treatment. Derivatives of three aniline sulfonic acids in the role of a dye were subjected to oxidative polymerization using potassium peroxydisulfate. The dyeing process was then performed with aniline-3-sulfonic acid as a polymer, aniline-4-sulfonic acid as a dimer, and 4-methylaniline-2-sulfonic acid as a trimer. The examination was focused on polymerization conditions as well as the influence of the parameters of the dyeing procedure on the resulting color properties of the wool, changes in the elemental composition of the dyed wool, infrared spectra, and the microscopic structure of the fiber surface. In contrast to the application of the dimeric and trimeric forms of the dye, a thin deposit of the dye on the fiber surface was observed only for the polymeric dye. Changing the processing parameters affected the resulting shade and intensity of the color. The optimum processing conditions were found to be pH 3, 30 °C, and 60 min. The energy balance of the energy consumption of the described dyeing process showed that, compared to conventional boiling dyeing, the energy consumption is less than 25%.

In the case of natural white wool, yellowing is observed after a certain exposure period to daylight. Paradoxically, when dyeing wool, we strive to change the original color in the technological process, but spontaneous color change, yellowing, is undesirable. It is caused by the ultraviolet component of UVB solar radiation (280–315 nm), which, even if only a small part (less than 3%) falls on the earth protected by the ozone layer, interacts with photoactive amino acid residues, including cystine, tryptophan, tyrosine, and phenylalanine [27]. The products of these interactions shift the absorption of electromagnetic radiation to the longer wavelengths of the visible spectrum. Yellowing can be limited by the addition of a UV-blocker, whose absorbance coefficient ε for the UV region is higher than that of keratin. The blocker thus preferentially absorbs UV energy and dissipates it in the form of harmless heat using an intramolecular mechanism. This will prevent UV-activation of reactions of photoactive components of keratin leading to yellowing of wool. Chen et al. [27] developed an eco-friendly reduction of yellowing of wool fabric by introducing p-aminobenzoic acid into keratin. They used the route of microbial transglutaminase via acyl transfer reaction in Tris-HCl buffer at mild conditions. The binding of p-aminobenzoic acid to wool by covalent bonds was verified using fluorescent and UV–Vis reflectance spectra. The thus-modified wool was subjected to the accelerated test of light stability using irradiation by a xenon arc lamp. At the optimal concentration of p-aminobenzoic acid, a dramatic improvement in the UV protection factor was found. In some cases, after prolonged irradiation of the modified samples, a certain improvement in mechanical properties was also observed, which can be attributed to some cross-linking of keratin macromolecules initiated by radicals created by the UV radiation effect. As shown by the SEM images of the irradiated samples, the applied p-aminobenzoic acid prevented the fiber surface erosion.

## 6. Modification of Sheep Wool

The effort to valorize wool also for non-textile applications has led to various physical and chemical treatments of wool. Researchers were inspired by the possibility of incorporating wool into composite materials with a matrix based on non-renewable resources (but not only) and thereby saving them. A decisive condition for success is good adhesion of keratin to the actual matrix, as it determines other important properties.

Competitive efforts in the area of reducing the prices of building materials find a response in the search for the possibility of incorporating sheep wool into mortar and concrete according to the purpose of use. Alyousef et al. [28] tested samples of standard concrete with an admixture of sheep wool with a uniform length of 70 mm in the amount of (0–6)% and samples with (0–1.5)% of modified wool. The presence of sheep fibers made the mixture difficult to process. The modification of wool consisted of a simple immersion of natural wool in a container filled with 35% saline solution for 24 h in order to increase the fiber surface friction and provide better bonding strength. The prepared specimens were tested after 7, 14, and 28 days of curing for strength, splitting tensile strength, flexural strength, and microstructure. As measured, the addition of wool fibers reduced the compressive strength a little. However, for samples with modified wool, the compressive strength was higher than that of untreated fibers at all curing times. For all concrete specimens, higher tensile and flexural strength was observed. Summarizing all results, around (2–3)% of natural sheep wool and (0.5–1)% of modified sheep wool seem to be the optimum levels in concrete. The inclusion of wool fibers in concrete also resulted in a better ductility of specimens and higher energy absorption [28].

Natural wool was also tested as a reinforcement in epoxy composites [29]. The matrix was a liquid epoxy resin based on bisphenol-A (4,40-isopropylidenediphenol, oligomeric reaction products with 1-chloro-2,3-epoxypropane) and cured after mixing the components. Prepared composites with a composition of epoxy resin/wool = 60/40 and 50/50 were subjected to tests including the tensile test, bending test, water absorption test, chemical absorption test, and biodegradable test of woven sheep fiber. Some results were better with the prevalence of epoxy resin and others with a balanced composition. Strength characteristics such as tensile and bending strength measured on 50/50 woven fiber composites were higher than for 60/40 composites. The results point to the fundamental influence of wool fibers in the thermoset matrix, which, unlike wool, is more rigid and less ductile. The wool acts as a reinforcement and contributes to maintaining the integrity of the composite, which is consistent with better results for the composite with a higher proportion of wool (50/50). The 50/50 sample absorbed more moisture than the 60/40, which, on the contrary, showed higher chemical absorption. In the biodegradability test, the samples were kept in the manure for a month and the significant decrease in their weight was almost identical. The resulting physical and chemical properties were related to the microstructure of the composites observed via scanning electron microscope.

Nitrate contamination of drinking water in wells is a common side effect of pollutants on smaller farms that raise animals. A simple modification of the wool by soaking it in a suitable acid can change the surface charge from a negative to a positive one. As shown by zero-charge point determination [30], the action of hydrochloric acid (0.01 M) or citric acid (0.1 M) on the wool led to the protonization of the wool. This created suitable conditions for electrostatic interaction with anions, which was demonstrated by the adsorption of nitrate anions onto the modified wool. The applied aqueous solution of calcium nitrate within nitrate concentrations of (20–100) mg/dm^3^ included the permitted limit of nitrates in drinking water of 50 mg/dm^3^. As shown by UV-spectroscopic measurements for λ = 232 nm, wool modified with 0.01 M hydrochloric acid provided better nitrate adsorption (up to 5 mg/g) than modified with 0.1 M citric acid (up to 1.7 mg/g). Among the eight tested adsorption isotherms, the Freundlich, Temkin, and Halsey models fitted best, indicating the inhomogeneity of the adsorbed layer [30].

The polar character of sheep fiber is the reason why, without proper treatment of the fiber surface, the adhesion to the non-polar surface of olefinic polymers such as polyethylene, polypropylene, or polystyrene is not sufficient. Unlike these polymers, poly(lactic acid) (PLA) has a polar character. Moreover, it is a biopolymer that can be synthesized from simple sugars obtainable from biomass. In addition, it has comparable physical properties to olefinic polymers, which favors it in the selection of potential matrices. Pawlak et al. [31] investigated the compatibility of PLA plasticized by means of melanized linseed oil with short fibers of sheep wool. Before mixing the fibers in the same amount of 1 phr into the PLA matrix, the fibers were modified with four different compatibilizers, namely (3-(2-aminoethylamino)propyl)-trimethoxysilane, trimethoxy (2-(7-oxabicyclo (4.1.0)hept-3-yl)ethyl) silane, tris(2-methoxyethoxy)(vinyl) silane, and titanium (IV) (triethanolaminate) isopropoxide. The surface modifications of the wool were examined using FTIR and TGA methods. The authors subjected the model composites to physical–mechanical tests, namely tensile, flexural, hardness Shore D and impact resistance Charpy. The results compared with the characteristics of the matrix with the unmodified fiber showed an improvement in the measured parameters, but each modification worked individually. The best combination of tensile properties was shown by the modification with titanium (IV) (triethanolaminate) isopropoxide, but the thermal stability decreased by 10 °C. The best Charpy impact strength was measured for the trimethoxy (2-(7-oxabicyclo(4.1.0)hept-3-yl)ethyl)silane modification. As can be seen from the DSC measurements, composites with modified fibers showed a higher crystallization temperature, a lower crystallization rate, and a lower crystallinity. This indicates lower mobility of the matrix macro-chains hindered by improved adhesion of the modified fibers to the matrix. The effect of a higher portion of wool (1–10 phr) modified with tris(2-methoxyethoxy)(vinyl) silane in a composite with the same plasticized PLA matrix was investigated in a follow-up study [32]. Testing included physico-mechanical, thermal, microstructural, and surface properties as well as coloring of the composite. FTIR spectra and SEM images confirmed the positive effect of the modification. While comparative samples of composites with untreated fiber generally achieved a decrease in mechanical properties, analogous composites with silanized surfaces slightly compensated for such a decrease. The loss of properties increased with increasing fiber content, but the 2.5 phr-modified wool sample showed the highest Young’s modulus and elongation at break. Charpy impact toughness and Shore D hardness were not statistically significantly different for individual formulations. The increasing wool content hydrophobized the composite and the yellow coloring of the material took on a darker shade.

Improvement of the interfacial adhesion of natural rubber latex in the rubberization of coarse wool fabric was achieved by Jose et al. using sodium ligno sulfonate (SLS) at different concentrations [33]. SEM images revealed that SLS fulfills the role of fabric sealant with latex. The interaction was manifested by an increase in area density of up to 32%. At low concentrations of SLS, the ductility of the composite increased considerably and, conversely, the diffusion of water was significantly reduced. It was shown that the UV-aged sample with a 50% concentration of the SLS better preserved the tensile strength. Variations of the formula can be used for several tailored applications with the required properties, e.g., imitation leather.

In the case of the polypropylene (PP) matrix, both a commercial type of PP and melanized PP were used [34]. PP composites with native wool, oxidized, and silanized wool were compared. Preoxidized wool was prepared by soaking in a preheated aqueous solution of hydrogen peroxide followed by heating. This increased the surface content of functional groups –SO_3_H and –COOH, which raised the reactivity of the wool in the next step. The silanizing of preoxidized wool fibers was performed using [3-(methacryloyloxy)propyl]trimethoxy silane. Composites containing 20% fibers were prepared by simply mixing 2 cm long fibers into the PP melt at 170 °C in an internal mixer (Brabender). Polypropylene-based composites were achieved, as confirmed by FTIR and SEM analyses. The FTIR spectra revealed structural changes related to the amount of bound silane. The SEM images of the fracture surface of the composite with silanized wool compared to the composite with untreated and oxidized wool showed improved fiber adhesion to the PP matrix, although not perfect. The wool treatment did not significantly affect the standard mechanical properties of the composite, but it had an effect on the wool breakage resistance under mechanical stress during the mixing of the components in the melt. The result of the microscopic analysis pointed to the different tendencies of the fibers to break and their resulting polydispersity. The composite with silanized wool achieved the best homogeneity and, significantly, the highest proportion of fibers with a supercritical length in the resulting composite.

It is known that wool has good not only thermal and acoustic insulation but also fire characteristics. The wool is self-extinguishing, the fibers do not support combustion, and they do not drip, but char at high temperatures. The ignition temperature of wool is 570–600 °C. Therefore, the idea of using wool as a flame retardant is not surprising. The study by Kim et al. [35] is devoted to the investigation of the flammability of PP-based composites with 30% wool and the addition of 20% fire-retardant ammonium polyphosphate. Model samples were prepared from two PPs with different melt flow indexes using a twin-screw extruder at (175–179) °C and subsequently pressed into test specimens. Thermal decomposition via thermogravimetric analysis and flammability tests, namely time to ignition, heat release rate, peak heat release rate, total heat release, and smoke production were tested as fire characteristics. Also, vertical burn tests to qualify burning characteristics such as self-extinguishing and sustained flame with dripping were observed. The flammability tests confirmed the improved fire retardancy. Moreover, the heat release rate and direct flame self-extinguishment of composites were significantly reduced. As expected, a higher melt flow index also positively affected the dispersal of ammonium polyphosphate and therefore flammability properties. The wool in the composite decreased polymer dripping, which reduces possible fire spread. Compared to PP, the composite with wool and ammonium polyphosphate showed improved tensile moduli.

As reported by Guo et al. [36], an efficient and durable flame retardant of wool fabrics can be relatively easily acquired by applying chelation of ferric ions. Activation of the wool fabric was performed by immersing it stepwise in a solution of NaClO and then in a solution of Na_2_CO_3_ and Na_2_SO_3_. Then came immersion in 6% FeCl_3_ at 50 °C for 50 min followed by curing in an oven at 80 °C for 10 min. The final samples were subjected to fire-technical tests. Measurements of limiting oxygen index (LOI) and cone-calorimeter tests (CCT) indicated that the ferric ions not only increased the LOI value from 24.2% to 35.3% but also compressed the peak heat release rate and total smoke release value. A high LOI value was observed even after 30 laundering cycles. The smoke density of the wool fabrics was reduced by 60.7%. The authors interpret the ferric ion to be a catalyzer of a carbon layer formation on the wool fibers at a lower temperature and being oxidized creates a Fe_2_O_3_ layer at higher temperatures. The carbon and Fe_2_O_3_ layers are a protective barrier against further burning and suppressing smoke leakage.

A demonstration that biobased materials have the potential to replace some non-renewable resources is also the case of an adhesive made from wool by its chemical transformation [37]. The preparation of the adhesive included the hydrolysis of wool by a microwave thermal process. The wool–hydrolysate (WH) was mixed with cationic polyamidoamine-epichlorohydrin (PAE) resins. As proved by FTIR, DSC, and TGA, cross-links were created between the carbonyl and amine groups of the components. The resulting adhesive was successfully tested on plywood sheets. The strength parameters were comparable to the commercial resin; nevertheless, soaking and boiling reduced the shear strength of WH-PAE composites. The advantage of the adhesive is, that unlike formaldehyde-based glues, the prepared adhesive is non-toxic.

Shahid-ul-Islam et al. [38] reported the modification of wool by the amino polysaccharide chitosan, as a green agent instead of toxic metal salt mordants in the pre-treatment step. Chitosan coated on the wool gave the wool an excellent color effect measured by CIEL*a*b* color parameters when dyed with tea extract polyphenols. The synergy of the dye and chitosan also showed radical scavenging and antibacterial properties in relation to *E. coli* and *S. aureus*. Analysis of the dyed wool using FTIR and thermogravimetry showed that the reason for the good affinity of the wool to chitosan is hydrogen bonding, electrostatic, and van der Waals interactions. As the authors state [38], the application of chitosan as a pre-treatment offers excellent opportunities for the development of multifunctional woolen fabrics in the field of dyeing with natural dyes and medical garments, hygienic clothing, sportswear, and undergarments.

The use of chitosan due to its advantages such as biodegradability and antibacterial effect is the subject of research by other researchers. Wang et al. [39] studied the improvement of the properties of wool by grafting chitosan onto wool via “one-enzyme double catalysis”. The goal was to functionalize wool fiber so that two-dimensional covalent multiple crosslinks between chitosan and wool were formed by laccase/2,2,6,6-tetramethylpiperidine-1-oxyl (TEMPO) mediated by oxidization. The result of the grafting was examined by UV, ATR-FTIR, GPC, MALDI-TOF MS, and SEM. Modified wool showed significantly better dimensional stability compared to felting (2.53%), wettability, and dyeability. The authors conclude that the shown path for the enzymatic modification of keratin fibers can be an inspiration for other natural biopolymers with a similar structure.

In general, grafting specific monomers onto wool fiber is a good tool for altering the properties of wool according to the intended use. Therefore, wool processors can develop new products based on renewable biomaterials changing the physico-chemical properties. Shavandi et al. [40] provided an overview of potential monomers, initiators of grafting reactions, reaction mechanisms, and the influence of important factors on grafting reactions such as time, temperature, pH, and the concentration of grafting initiators. However, it should be kept in mind that in some cases the grafting of a specific monomer may impair other important application properties of the resulting copolymer. Also, the related chemical process may produce dangerous by-products and contaminate water. The source of hazardous waste can be unreacted monomers or also initiators. These include metal chelating ions such as quinquevalent V, Cr(VI), Cu(II), benzoyl peroxide, peroxydiphosphate, peroxydisulfate, bromide salts, and others [40].

Nikiforova et al. [41] studied the grafting of wool on keratin side chains with which they wanted to improve the sorption properties of Cu(II) on wool. For grafting, they used a vinyl monomer in the form of acrylamide, in which they first substituted the carbamide group with the hydroxamic one. The modified monomer CH_2_=CH-CO-NHOH was subjected to a polymerization reaction with the Wool-S* radical created by the action of the Fe(II) redox system as an initiator. In the grafted keratin, the original surface carboxyl groups were preserved and acidic hydroxamic groups were added. The grafting process can be described by Equations (1)–(3).

(1) $$Wool-S-S-Wool\overset{{Fe}^{2+}}{\to }2\;Wool-S^{*}\;$$

(2) $$Wool-S^{*}+nCH_{2}=CH-CONHOH\to Wool-S-[C_{2}H_{3}\left(CONHOH\right)-{]}_{n}-CH_{2}C^{*}H\left(-CONHOH\right)\;$$

(3) $$Wool-S-[C_{2}H_{3}\left(CONHOH\right)-{]}_{n}-CH_{2}C^{*}H\left(-CONHOH\right)+R^{*}\to Wool-S-[C_{2}H_{3}\left(CONHOH\right)-{]}_{\left(n+1\right)}-R$$

As confirmed by FTIR spectroscopy and SEM microscopy, this was a consequence of the threefold increase in Cu(II) binding.

**Figure 4:**
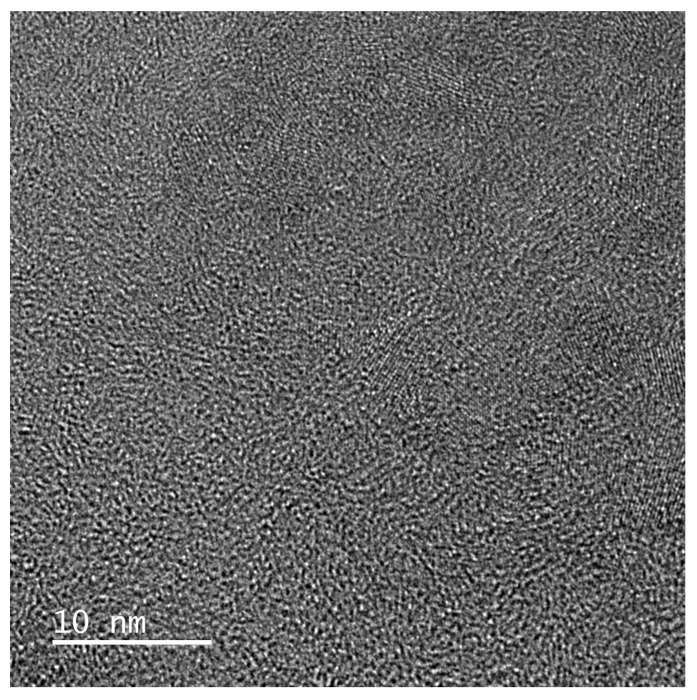
SEM photo of biochar powder from US scoured wool after heating to 1400 °C (with the kind permission of M. Čaplovičová).

Carbon fibers as a component of various composites have been a hit in the last few decades because, depending on the raw material and the technology of preparation, they have various interesting properties. The C-fiber is a continuous fiber containing a massive majority of carbon atoms having a diameter between 5 and 10 μm. The greatest advantages of C-fibers are excellent structural properties, superlative ratio of tensile strength to weight, and thermal and chemical resistance. In the manufacture of carbon fiber, carbon atoms are combined into a crystal lattice parallel to the longitudinal axis of the fiber, which results in high tensile strength. These properties, together with the very low weight, predetermine it as an excellent construction material in aeronautical and space technology, in sports facilities, motorsports, and the army. The first fibers were made by sintering a bundle of viscose fibers until they were carbonized, and later polyacrylonitrile fibers were used as the source material. The question is whether protein keratin containing nitrogen in the backbone chain can be a suitable and cheap precursor. In this sense, works appear that be an interesting source of information. Pina et al. [42] published a study on thermal–oxidative stabilization of wool fibers when heated to (250–300) °C. The goal was to preserve the fibrous character of the wool even at higher temperatures. As shown, upon heating, sulfur and oxygen groups decreased, S-bridges were cleaved, and oxidative cross-linking was observed. The amide groups were transformed into carbodiimide groups RN=C=NR depending on temperature and time, but below 400 °C the primary wool structure and polypeptide bond were preserved. While virgin wool, converted to dry ash, contained 49 wt. % C, with increasing heating temperature this portion increased and at a temperature of 300 °C recorded a content of 62 wt. % C. This knowledge is valuable for further research on the potential preparation of carbon fibers from waste wool. Figure 4 shows SEM photos of biochar prepared from wool fiber. After heating wool up to 1400 °C, any fiber structure is not visible.

### Modification of Wool Using Radiation Technologies (Corona, Plasma, Electron Beam)

As already mentioned, the hydrophobic nature of wool makes it difficult to adhere to more polar materials. On the other hand, thanks to this property, sheep do not become wet in the rain, even though the wax (lanolin) naturally covering the surface of the fibers contributes most to the water repellency of raw wool. When combining materials with a more hydrophilic matrix than wool, the reduction of hydrophobicity of wool and thus better adhesion of wool can be achieved by applying radiation methods to the fiber surface. It is corona discharge [43,44] or plasma [45]. These radiation techniques are more environmentally friendly because they are dry, waste-free processes.

The effect of surface modification of wool fabric on the dynamic heat and mass transfer was investigated by Li et al. [46]. To modify the fabric, they used corona discharge (6 kW glow discharge [47], voltage of 12 kV) and hydrogen peroxide oxidation (12% solution, temperature 65 °C) individually as well as their joint combination performed in alternating order. As confirmed by SEM images, both procedures induced cuticle disruption. The etching effect of the corona and oxidation with H_2_O_2_ led to the breaking of the scales, their rounding, or even peeling. The contribution of chemical oxidation by peroxide to the surface change appears to be greater than the physico-chemical effect of the corona. As a result of the cracks in the cuticle, the modified surface of the fiber gained greater wettability and absorptivity. This was confirmed by the reduction of the contact angle due to the increase of oxygen and the decrease of S-S groups on the surface. Better absorptivity can be used when making sports clothes that absorb sweat better and faster. The change in thermal conductivity, lower surface temperature, and less energy consumed in contact with a rotating plate heated to 35 °C indicated better heat retention by such modified woolen fabrics. At the same time, slower evaporation of moisture was found in the modified samples because it is more strongly bound on the wool surface by the formed polar groups. Knowledge from this work can be used in the development of functional clothing with better comfort for the user.

To improve the hydrophilic properties of wool fabric, Wang et al. [43] combined corona discharge with a hydrogen peroxide treatment. Using SEM, a corona etching of the tip of wool scales was observed and, after the hydrogen peroxide treatment, the scales were peeled off partially. A combination of those two effects resulted in absolutely hydrophilic wool fabric. However, the fabric became weaker and more flexible with an average weight loss of 3%.

Xu et al. [44] applied corona discharge on wool fabrics at different experimental conditions and investigated the effect of corona on the fabric resistance to shrinkage. Further development showed that it is still possible to improve it enzymatically or by finishing with resins.

Kan and Yuen [45] investigated the effect of the low-temperature plasma on wool fibers using different gases, namely oxygen, nitrogen, and a gas mixture (25% hydrogen/75% nitrogen). Using FTIR-ATR, XPS, and Methylene Blue adsorption, they analyzed changes in the surface structure. The chemical composition of the surface of the plasma-treated wool varied depending on the gas. The content of nitrogen and oxygen increased, while the representation of carbon and sulfur decreased. It was found that sulfur was oxidized from S(II) to S(VI) and the surface area increased significantly. These changes improve the hydrophilicity and fundamentally increase the reactivity of the wool. They can positively affect the dyeability, finishing, and shrink-proofing of the wool. Since the process is performed in a plasma chamber under vacuum or in an atmosphere of a selected gas, it cannot be continuous in general, but batch-like.

The development of radiation technologies made it possible to extend the treatment of materials with an accelerated electron beam. The advantage of this technique is that, unlike corona discharge or plasma, the electron beam penetrates the entire volume of the fiber. This process can be implemented as a continuous process even on an industrial scale.

The first report on the effect of the electron beam on sheep wool was presented by Porubská et al. [48]. In the detailed study, they described how S-oxidized species, -CH groups, secondary structure, temperature and enthalpy of crystal cleavage, strength, and elongation of the fibers changed according to the absorbed dose of energy in the observed range of absorbed doses of (0–400) kGy. The original portion of the α-helical structure changed to β-sheet conformation with increasing dose. Keratin disulfide bridges were broken and oxidized in the air via S-sulphonate, cystine monoxide, and cystine dioxide to the final cysteic acid as Equation (4) shows:

(4) $$R-S-S-R\overset{e}{\to }R-S^{\left(-\right)}\dot{-}S-R\overset{O_{2}}{\to }R-S-SO_{3}^{-};R-S-SO-R;R-S-SO_{2}-R;R-SO_{3}^{-}$$

**Figure 5:**
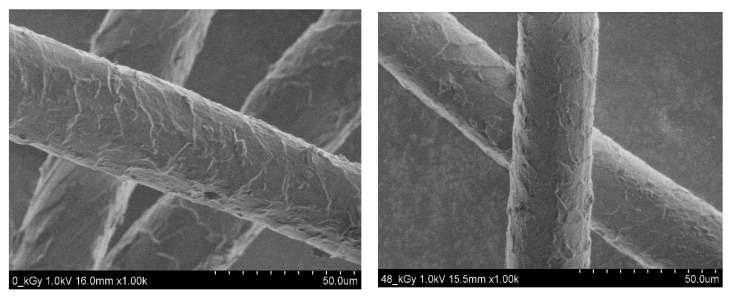
SEM photos of US scoured non-irradiated (**left**) and US scoured and irradiated (**right**) fibers (under CC license).

Higher doses around 200 kGy caused easy cross-linking of the chains through hydrocarbon groups, and around (300–400) kGy the keratin chains already started to tear. The surface appearance of US-scoured fiber before and after being irradiated is displayed in Figure 5.

**Figure 6:**
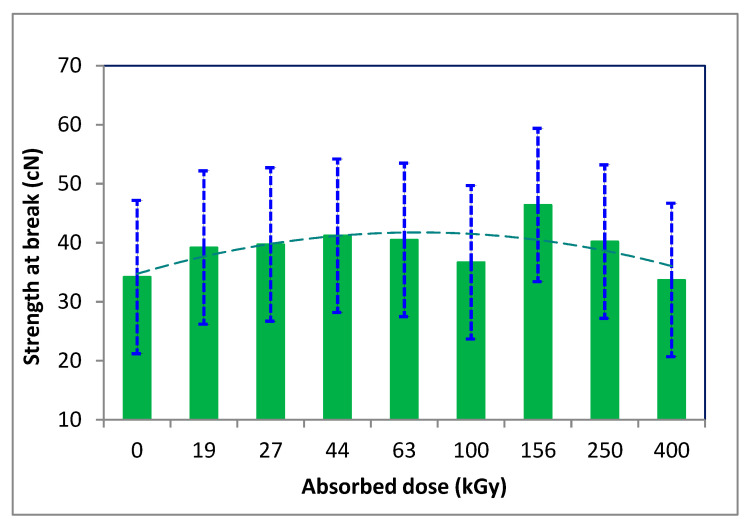
Variation of tensile strength at break of the irradiated wool depending on absorbed dose (adapted from [48]).

**Figure 7:**
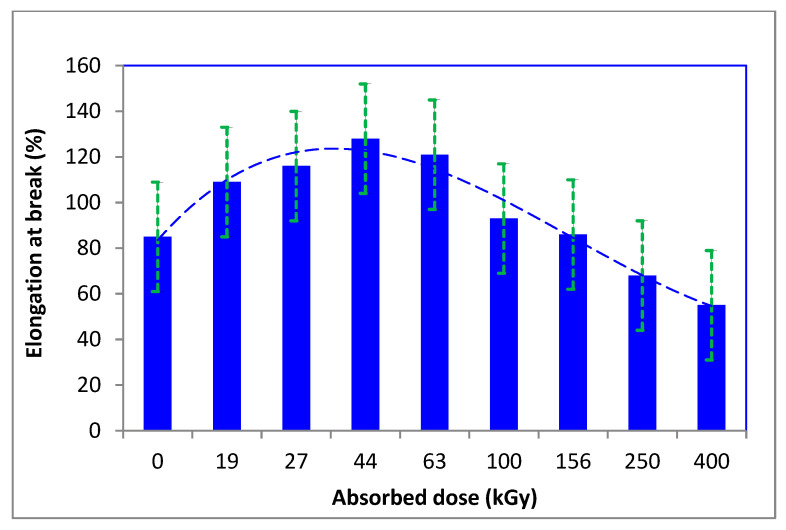
Variation of elongation at break of the irradiated wool depending on absorbed dose. (adapted from [48]).

The strength and elongation of the fibers did not change significantly; after an initial increase, they gradually decreased with an augmenting dose of absorbed energy (Figure 6 and Figure 7).

**Figure 8:**
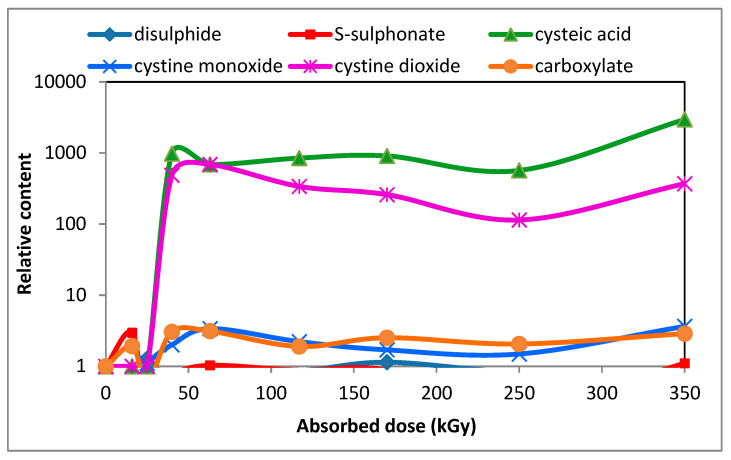
Development of relative content of cystine dioxide and cysteic acid along other species in the wool with absorbed dose 88 days after electron beam irradiation (under a CC license [49]).

An interesting finding was the time-dependent increase in the content of cysteic acid [49] as can be seen from Figure 8, although 2 days after irradiation the cysteic acid content was barely measurable.

Although keratin has interesting adsorption properties due to various functional groups in the side chains, in the irradiated wool, cysteic acid was added to the carboxyl groups. This provided a prerequisite for improved interaction of the irradiated samples with metal cations, including chemisorption. The hypothesis was confirmed in subsequent papers [50,51]. According to their chemical nature, metal cations form complex salts with keratin (carboxylates, cysteinates) with ligands originating from the side functional groups of keratin having a free electron pair [51]. The adsorption capacity of the wool can be regulated, among other effects, by the dose absorbed when the wool is irradiated in an electron accelerator [52].

The potential of electron-modified wool awaits practical application as a cheap adsorbent in the field of pollutant elimination, especially, but not only, in the aquatic component of the environment.

## 7. Overview of Topics Covered Study

An overview of the topics covered in this review can be seen in Table 2.

## 8. Conclusions

The achieved results brought new knowledge in each analyzed area. However, their practical application is usually a lengthy process. The development of meaningful and effective use of sheep wool will support the development of sheep farming, contribute to the alleviation of food shortages, provide job opportunities even for less qualified people, and help to improve landscaping. Practical life can bring other directions of research and development that cannot yet be defined today.

The expected directions of research and development in the innovative use of sheep wool may include the following: the development of scouring lines without chemicals based on ultrasound, the modification of wool by an accelerated electron beam, the research of other composites containing wool for both interior and exterior applications, and the design of technology to incorporate wool into different matrices, achieving good homogeneity. Research should also include opportunities for the elimination of environmental burdens with the use of purposefully modified wool, improving the resistance of wool to harmful insects for the purposes of thermo-acoustic insulation of buildings, air purification in smoking interiors, elimination of the so-called welding smog in the working environment, valorization of unusable wool waste in the agricultural sector, including controlled biodegradation in the soil, and more. Each of the mentioned aspects can be realized in start-ups.

## Figures and Tables

**Table 1:** Relative loss of wool mass [11].

Laboratory Scouring Procedure	Relative Loss of Wool Mass (%)			
	First Cycle	Second Cycle	Third and Fourth Cycles	Total Loss
US (2 × 10 min)	6.8	5.0	negligible	11.8
US (3 × 10 min)	7.1	5.1	negligible	12.2
US (1 × 20 min)	6.7	4.8	negligible	11.5
US (1 × 30 min)	7.1	4.9	negligible	11.0
Soxhlet Extraction	10.0	-	-	10.0

**Table 2:** Summary of the topics covered.

Section	Topic	Authors
3	***Ultrasound scouring***	
	Effect of US frequency and power	Li et al. [8]
	Effect of US scouring on color strength of dyed wool	Pan et al. [9]
	Effect of US scouring on color strength of dyed wool	Nasmul Islam et al. [10]
	Comparison of the scouring using US bath with and without detergent and using Soxhlet dichlomethane extraction	Braniša et al. [11]
	Two-phase scouring; gross impurities in water bath and US bath with detergent, alkali soap, and sodium carbonate.	Czaplicki et al. [12]
	Effect of US bath temperature on dyeing	Grande et al. [13]
	Effect of US bath on wool morphology, physico-mechanical characteristics, and dyeability	Pan et al. [14]
	Use of immobilized thermophilic lipase to remove the lipid layer	Taleb et al. [15]
4	***Anti-felting and anti-shrinking treatment***	
	Application of biosurfactant	Iglesias et al. [16]
	Enzymatic treatment, radiation, plasma,	Hassan et al. [17]
	Use of biopolymers (gum arabic, chitosan, and wheat starch)	Rani et al. [18]
	Enzymatic cleavage of the cuticle	Kadam et al. [19]
	Hydrolysis wool scales by protease	Li et al. [20]
5	***Dyeing of wool***	
	Improvement of color printing	An et al. [21]
	Improvement of color printing	Wang et al. [22]
	Dyeing using oxygen plasma	Barani & Haji [23]
	Dyeing applying dye synthesis	Hassan et al. [24]
	Dyeing using grafting	Sadeghi-Kiakhani et al. [25]
	Dyeing using oxidative polymerization	Hassan [26]
	Reduction of yellowing of wool fabric	Chen et al. [27]
6	***Modification of sheep wool***	
	Modification of concrete by adding sheep wool	Alyousef et al. [28]
	Composite epoxy-resin with sheep wool	Bharath et al. [29]
	Acidic modification to adsorb nitrates	Porubská et al. [30]
	Wool modification using compatibilizers	Pawlak et al. [31]
	Wool modification using silane	Pawlak et al. [32]
	Rubberization of wool fabric	Jose et al. [33]
	PP composite with silanized wool	Conzatti et al. [34]
	Fire retardant for PP-based composite with wool	Kim et al. [35]
	Fire retardant of wool applying chelation of ferric ions	Guo et al. [36]
	Adhesive from wool hydrolyzate	Shavandi & Ali [37]
	Chitosan coated on wool	Shahid-ul-Islam et al. [38]
	Grafting of chitosan onto wool	Wang et al. [39]
	Grafting of specific monomers onto wool	Shavandi & Ali [40]
	Grafting of modified acrylamide onto wool	Nikiforova et al. [41]
	Thermal-oxidative stabilization of wool heated	Pina et al. [42]
	Corona discharge with hydrogen peroxide treatment	Wang et al. [43]
	Corona discharge modification	Xu et al. [44]
	Low-temperature plasma effect on wool	Kan & Yuen [45]
	Corona effect on wool	Li et al. [46]
	Corona effect on wool and hydrogen peroxide oxidation	Li et al. [47]
	Effect of the electron beam on sheep wool	Porubská et al. [48]
	Time-dependent structural variations wool irradiated by electron beam	Hanzlíková et al. [49]
	Effect of the electron beam on sorptive properties of wool	Hanzlíková et al. [50]
	Atypic sorption of Cu on irradiated wool	Porubská et al. [51]
	Competitive sorption of metals on irradiated wool from binary solutions	Braniša et al. [52]

## Data Availability

Data are contained within the article.
